# Estimating Litter Decomposition Rate in Single-Pool Models Using Nonlinear Beta Regression

**DOI:** 10.1371/journal.pone.0045140

**Published:** 2012-09-25

**Authors:** Etienne Laliberté, E. Carol Adair, Sarah E. Hobbie

**Affiliations:** 1 School of Plant Biology, The University of Western Australia, Crawley, Western Australia, Australia; 2 National Center for Ecological Analysis and Synthesis, University of California Santa Barbara, Santa Barbara, California, United States of America; 3 Department of Ecology, Evolution and Behavior, University of Minnesota, Saint Paul, Minnesota, United States of America; DOE Pacific Northwest National Laboratory, United States of America

## Abstract

Litter decomposition rate (*k*) is typically estimated from proportional litter mass loss data using models that assume constant, normally distributed errors. However, such data often show non-normal errors with reduced variance near bounds (0 or 1), potentially leading to biased *k* estimates. We compared the performance of nonlinear regression using the beta distribution, which is well-suited to bounded data and this type of heteroscedasticity, to standard nonlinear regression (normal errors) on simulated and real litter decomposition data. Although the beta model often provided better fits to the simulated data (based on the corrected Akaike Information Criterion, AIC*_c_*), standard nonlinear regression was robust to violation of homoscedasticity and gave equally or more accurate *k* estimates as nonlinear beta regression. Our simulation results also suggest that *k* estimates will be most accurate when study length captures mid to late stage decomposition (50–80% mass loss) and the number of measurements through time is ≥5. Regression method and data transformation choices had the smallest impact on *k* estimates during mid and late stage decomposition. Estimates of *k* were more variable among methods and generally less accurate during early and end stage decomposition. With real data, neither model was predominately best; in most cases the models were indistinguishable based on AIC*_c_*, and gave similar *k* estimates. However, when decomposition rates were high, normal and beta model *k* estimates often diverged substantially. Therefore, we recommend a pragmatic approach where both models are compared and the best is selected for a given data set. Alternatively, both models may be used via model averaging to develop weighted parameter estimates. We provide code to perform nonlinear beta regression with freely available software.

## Introduction

Litter decomposition strongly influences carbon and nutrient cycling within ecosystems [1]. Therefore, estimating an accurate decomposition rate is critical to understanding biogeochemical processes. The most widely used model to describe the rate of litter mass loss is the single-pool negative exponential model [2]

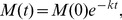
(1)where *M*(*t*) is litter mass at time *t*, *M*(0) is initial litter mass, and *k* is the litter decomposition rate. Because *M*(0) is generally known, its estimation is unnecessary and can even lead to biased estimates of *k*, the parameter of interest [3]. Thus, *M*(*t*) is best divided by *M*(0) and the resulting proportional litter mass loss *X*(*t*) modeled as [3]



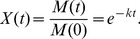
(2)In theory, *X*(*t*) is bounded such that 0≤ *X*(*t*) <1, but in practice values ≥1 sometimes result, especially during the early stages of decomposition.

**Figure 1:**
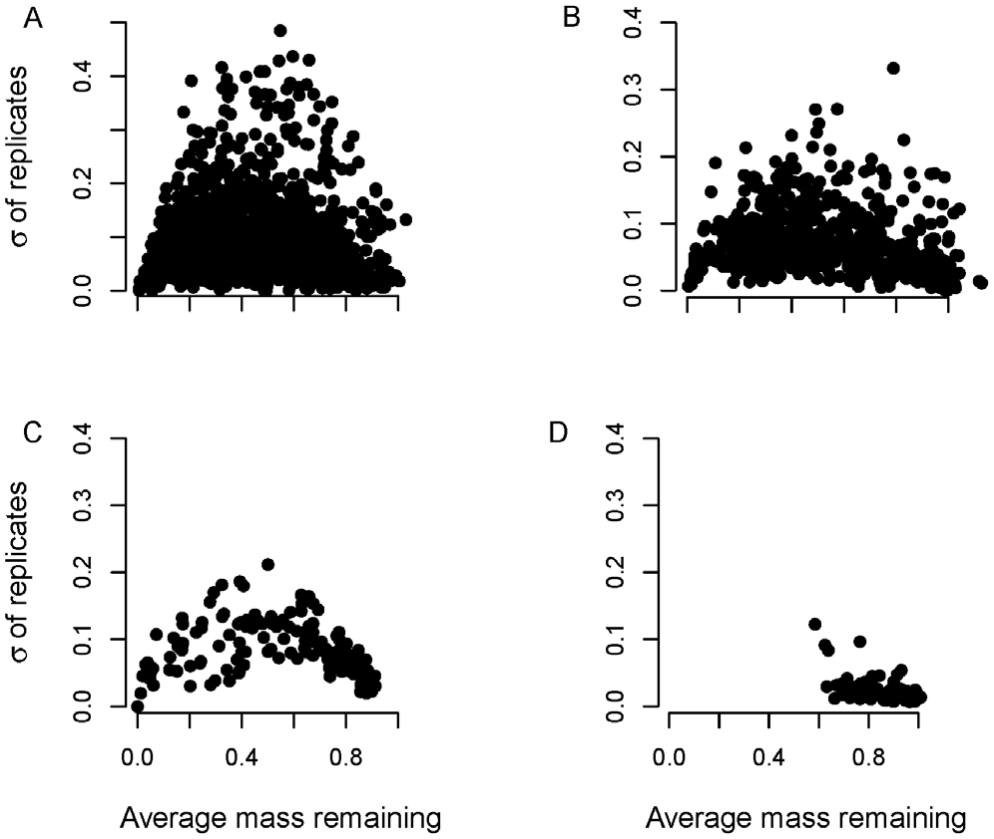
Figure of mean mass remaining versus standard deviation of replicates at each time point for real data. Mean mass remaining versus standard deviation of replicates at each time point for (A) Long-term Intersite Decomposition Experiment Team (LIDET) data, (B) Hobbie data; (C) EL data; and (D) HG data.

Often, *k* is estimated by log-transforming *X*(*t*) and using a linear regression model with mean *μ* and normally distributed errors, where *k* is the slope and *σ* ^2^ is the variance
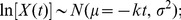
(3)this is similar to the use log-log regression for fitting allometric power equations [4] and biological power laws [5]. However, [3] showed that this approach leads to biased *k* estimates unless errors are log-normally distributed. Instead, they suggested using nonlinear regression on untransformed data, again with normally-distributed errors



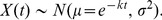
(4)This model was found to give more accurate *k* estimates in simulations [3], but it assumes that errors are constant and normally distributed – a likely invalid assumption (Figure 1). Indeed, proportional litter mass loss data often shows smaller variance near bounds (0 and 1), which is typical of bounded data [6]. In these cases, fitting a model with constant normal errors may lead to biased *k* estimates.

One solution could be to model the variance *σ* ^2^ as a function of *t*, but this requires additional parameters. An alternative solution may be to use an error distribution better suited to bounded data, such as the beta distribution [6]. Like the normal distribution, it only has two parameters. Unlike the normal distribution, it is bounded between 0 and 1, and can easily accommodate the type of heteroscedasticity shown in Figure 1 [6]. Its probability density function is a function of two scale parameters, *α* and *β*

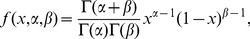
(5)where Γ(*n*) is the gamma function 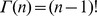 and 0≤*×*≤1. In the context of regression, the beta distribution is re-parameterized [6],[7] to a location parameter *μ* (the mean) and a precision parameter *φ* (the inverse of dispersion)
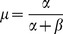
(6)

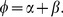
(7)


The variance *σ* ^2^ depends on *μ* and *φ*

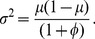
(8).

Consistent with patterns often found in decomposition data (Figure 1), the numerator shows that *σ* ^2^ is smaller near the bounds (0 or 1): if *φ  = *1 and *μ* = 0.01 or 0.99, *σ* ^2^ = 0.005; if *μ* = 0.5, *σ* ^2^ = 0.125. The denominator shows that higher precision *φ* reduces *σ* ^2^.

In summary, the beta distribution may be better suited than the normal distribution to model proportional litter mass loss data because it is bounded between 0 and 1, its *σ* ^2^ is smaller near its bounds, as with decomposition data (Figure 1), and hence it can model this type of heteroscedasticity without additional parameters.

Since the beta distribution is bounded between 0 and 1, proportional litter mass loss data must also be bounded between 0 and 1. However, litter mass loss data often contain values equal to 0 (no mass remaining), or ≥1 (no decomposition or sample contamination by soil), so the data, *y*, must be compressed to the ]0, 1[interval (*y*′′) [6]:
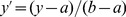
(9)

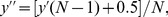
(10)where *a* and *b* are the *y* minimum and maximum values, respectively, and *N* is sample size. Hereafter, we refer to this transformation as Smithson and Verkuilen’s [6] (SV) transformation.

The goal of this paper is to compare the normal model (Equation 4) with the beta model
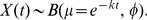
(11)Specifically, we : (1) compare the performance of the normal vs. beta model in numerical simulations, using different realistic error structures for simulated *X*(*t*); (2) investigate the influence of two different transformations to compress *X*(*t*) between 0 and 1, namely (i) treating zeros as missing data and setting values ≥1 equal to 0.9999, or (ii) Smithson and Verkuilen’s transformation [6]; and (3) compare the performance of the normal vs. beta model and evaluate the influence of the transformations mentioned above, using real data from decomposition studies of differing decomposition stage (early, medium, and late based on percent of initial mass remaining: 25, 60, and 72% average mass loss, respectively). Because different decomposition stages encompass different portions of the mean-variance relationships seen in litter decomposition data (Figure 1), we expected that it could influence the fit of beta vs. normal models.

We hypothesized that nonlinear beta regression would provide better fits to proportional mass loss data and give more accurate *k* estimates than normal nonlinear regression, because of the heteroscedasticity often associated with these data (Figure 1). If so, nonlinear beta regression would provide more reliable *k* estimates from single-pool models [2].

## Materials and Methods

### Data Simulation

We simulated *X*(*t*) using four values that spanned the range of low to high decomposition rates: 0.0005, 0.002, 0.01, and 0.1 d^−1^. These *k* values were chosen by examining the range of *k* values found in the Adair et al. [3] decomposition review and choosing values that spanned the range from very low to high (Figure S1). The chosen *k* values resulted in 1% mass remaining at approximately 25, 6, 1.3, and 0.1 years, respectively (using Equation 2; Table 1). We used these *k* values to simulate *X*(*t*) over four different time spans that represented early (80% mass remaining), mid (50% mass remaining), late (20% mass remaining), and end (1% mass remaining) stage decomposition for each *k* value (Table 1). This strategy allowed us to investigate the ability of each regression type to accurately predict *k* across a range of *k* values and decomposition stages (i.e., study lengths or total times).

**Table 1:** Percent mass remaining at early, mid, late and end stage decomposition for four different decomposition rates (*k* in d^−1^).

		Time (d)			
Stage	Mass remaining	*k = *0.0005	*k* = 0.002	*k = *0.01	*k = *0.1
Early	80%	446	112	22	2
Mid	50%	1386	347	69	7
Late	20%	3219	805	161	16
End	1%	9210	2303	461	46
Years to end		25.2	6.3	1.3	0.1

Time is the number of days (d) it takes for mass remaining to reach 80, 50, 20 or 1% for early, mid, late or end stage decomposition, respectively. Time is also provided in years for end stage decomposition.

To investigate whether the number of mass loss measurements taken within a given study would affect a given regression type’s ability to accurately estimate *k*, we generated 2, 5, 7 or 10 “measurements” across each *k* value and decomposition stage simulation. Because sampling times in decomposition studies are not typically evenly spaced, but are instead weighted towards the beginning of the study (where litter mass loss is most rapid), we used the data gathered during the review completed by Adair et al. [3] to determine sampling times: we (1) recorded total experiment time and all measurement times from each of the 383 references contained in the review; (2) converted measurement times to proportion of total experiment times; (3) grouped proportional measurement times by the number of times each study made mass loss measurements (i.e., 2, 5, 7 or 10 times); (4) created histograms for each category using bin sizes of 0.1; and (5) selected the most frequent proportional measurement times from each category (2, 5, 7, or 10 measurements; Figure S2). The proportional times used were the averages of the most frequent proportional measurement bins. Thus, for 2 measurements, data was simulated at 0.5 and 1.0 of total time (i.e., at ½ of the total time and at the end of the total time). For 5 measurements, data was simulated at 0.06, 0.14, 0.23, 0.63, 1.0 of total time. For 7 measurements, data was simulated at 0.05, 0.15, 0.24, 0.36, 0.54, 0.65, 1.0 of total time. For 10 measurements, data was simulated at 0.04, 0.11, 0.23, 0.32, 0.43, 0.53, 0.62, 0.84, 0.93, 1.0 of total time.

**Figure 2:**
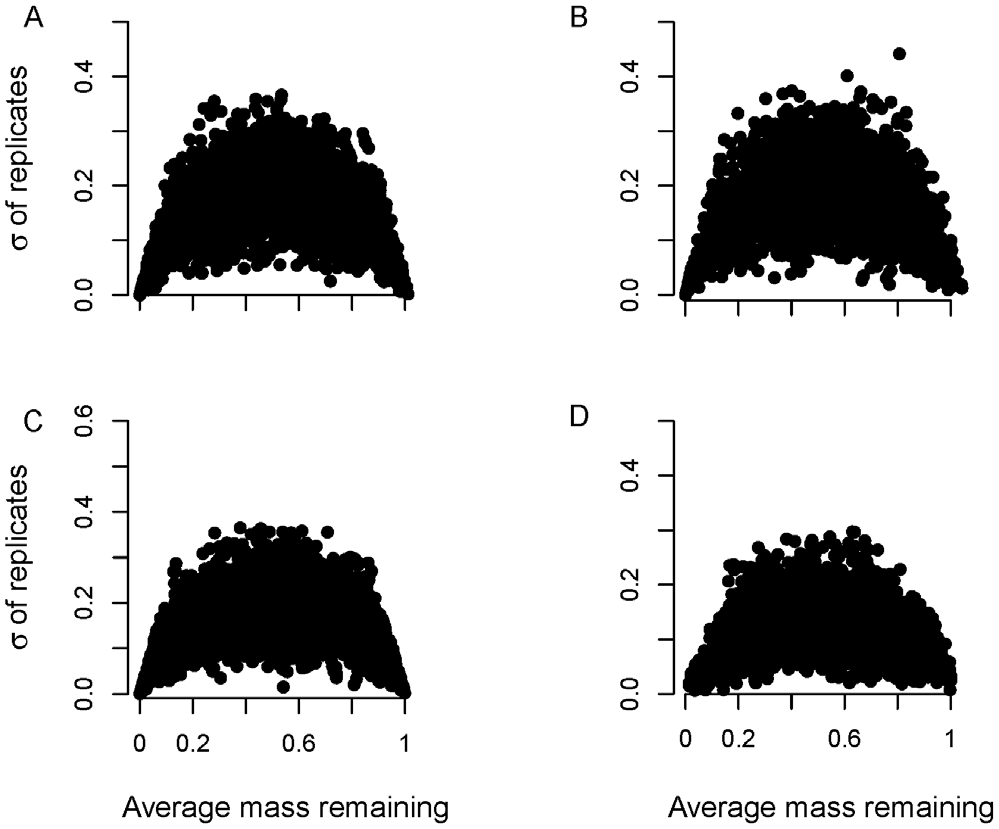
Figure of mean mass remaining versus standard deviation of replicates at each time point for 200 simulations with four different error structures: (A) beta errors + normal errors (option 3a; *σ* = 0.0125, *φ* = 5); (B) beta errors + normal error (option 3b; *σ* = 0.05, *φ*  = 5); (C) beta errors (option 2; no 0 or >1 values, *φ*  = 5); and (D) normal error with variable *σ* (option 1; Var *σ*_2_).

Finally, we used three different error structures that resembled those found in real data (Figures 1–2). For each error structure we generated data using three different standard deviations (or *φ’s* for beta regression) that resulted in low, moderate, and high variation in the simulated *X*(*t*)s:

#### 1. Normally distributed errors with variable standard deviations (σ)

We took random samples from the normal distribution
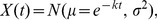
(12)using three different variable σ structures:
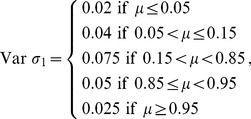
(13)

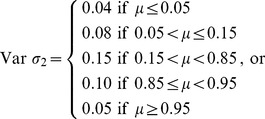
(14)

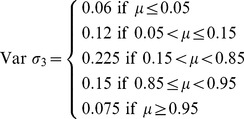
(15)


where σ increases from Var *σ*_1_to Var *σ*_3_. Values *X*(*t*) <0 were set to 0, whereas values *X*(*t*) >1.05 were set equal to 1 (Figure 2d).

#### 2. Beta-distributed errors

We took random samples from the beta distribution, with *φ* = 5, 8 or 15 (higher values generate less variation in *X*(*t*); Figure 2c).
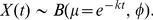
(16)


#### 3. Beta-distribution errors with normal errors added

We sampled from the beta distribution (*φ* = 5, 8 or 15) and added small amounts of normal error (*ε*; two different *σ* values) to generate values *X*(*t*) ≤0 or ≥1, which sometimes occur in real data.
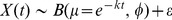
(17)where *ε* was either



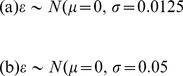
(18)Values <0 were then set equal to 0 (Figure 2a–b).

In total, we ran 768 simulations (four *k* values; three error options with three variable σ structures for option 1, three *φ* values for option 2 and six σ + *φ* combinations for option 3; four decomposition stages; four numbers of measurements). Each data set generated within a simulation run had five replicates per measurement time. We generated 12,000 data sets in each simulation run. We estimated parameters via maximum likelihood (ML) estimation with normal and beta distributed errors, using the ‘bbmle’ package (version 1.0.4.1) [8] and nonlinear least-squares regression (NLS; assumes normal errors), using the ‘nls’ function in R 2.15.0 [9]. At times, NLS and beta ML regression failed to converge. Thus, to compare regression methods, we used the first 10,000 simulations where all regression types successfully estimated *k*. NLS only failed in cases where simulated data sets contained many missing values (see REP transformation below). However, beta ML regression often failed to converge during early decomposition, regardless of the number of measurements that were used (2, 5, 7 or 10) to estimate *k*. This was especially true in simulations that used only beta-distributed errors (option 2). In these cases, we used <10,000 simulated data sets to compare regression methods (Table 2).

**Table 2:** Simulations for which ML estimation with beta errors (option 2) failed to converge for 10,000 out of 12,000 generated data sets.

			k = 0.1			k = 0.01			k = 0.002			k = 0.0005		
Error	Stage	# meas	*φ* = 5	*φ* = 8	*φ* = 15	*φ* = 5	*φ* = 8	*φ* = 15	*φ* = 5	*φ* = 8	*φ* = 15	*φ* = 5	*φ* = 8	*φ* = 15
Beta only														
	Early	5	5690	9784		6906			7123			6942		
	Early	7	4325	8519		5168	9351		5367	9517		5281	9409	
	Early	10	2581	6638		3274	7454		3390	7802		3335	7641	

The number of generated data sets for which ML estimation with beta errors converged is shown.

Additionally, when using ML estimation with beta errors to estimate the low *k* value (0.0005 d^−1^), optimization algorithms often failed to converge. We therefore estimated the low rate as a yearly rate (this solved the convergence problems) and converted it back to a daily rate for analyses, figures and tables.

For simulation runs that generated data sets with values *X*(*t*) = 0 or ≥1 (options 1, 3a, 3b), we compared two data transformations: the Smithson and Verkuilen (SV) [6] transformation (Equations 9 and 10) or, following [3], converting all values ≥1 to 0.9999 and treating zeros as missing data (the ‘replacement’ or REP transformation). For simulations with values 0< *X*(*t*) <1 (option 2), no transformations were necessary. This resulted in 576 additional simulations, for a total of 1344 simulations.

Because the generated data sets had small sample sizes (i.e. *N*/*p*<40, where *p* is the number of parameters), which is typical for litter decomposition studies, we used the corrected Akaike Information Criterion (AIC*_c_*) to compare models fitted via ML
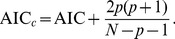
(19)


To determine how well the different approaches estimated the litter decomposition rate, *k_e_*, relative to the true *k_t_* (here, 0.002 d^−1^) we calculated the average percent (%) bias
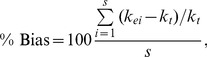
(20)where *s* is the number of simulations (here, *s* = 10,000 or as in Table 2), and the percent relative error (%RE)



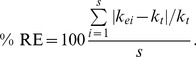
(21)Bias measured whether a particular approach over- or under-estimated *k_t_*, whereas %RE measured the magnitude of the difference between *k_t_* and all *k_e_*, regardless of direction.

Because results (% bias, % RE, and average *k* estimates, and AIC_c_ results) were very similar among *k* values (e.g., Figures S3,S4,S5,S6,S7,S8,), we present results from one *k* value (*k = *0.0002).

### Analysis of Real Decomposition Data

We used three real data sets that reflected the range of time frames used in the data simulation: early, mid, and late stage decomposition data, based on the proportion of initial litter mass still present at the end of each study (Table 1).

For the early stage decomposition data set, we used the Hobbie and Gough [10] litter bag decomposition data set. The average percent of initial mass remaining at the end of this experiment was 75.4% (standard error, SE = 3.1%), indicative of early decomposition. The Hobbie and Gough [10] experiment was conducted at two arctic tundra sites near Toolik Lake, Alaska (68 38′N, 149 43′W). Mean annual temperature (MAT) at Toolik Lake is −7°C with low annual precipitation (200–400 mm) [11]. In this experiment, nine litter types were decomposed over 1082 days. Five bags of each litter type were collected from each site on days 308, 361, 717, and 1082. Experiment details are presented in [10].

Mid stage decomposition data were provided by Laliberté and Tylianakis’ [12] 560-day litter bag decomposition experiment conducted on the AgResearch Mount John trial site, in the Mackenzie Basin of New Zealand’s South Island (43°59′S, 170°27′E). The climate is semi-continental with a MAT of 8.7°C and mean annual precipitation (MAP) of 601 mm. Litterbags of mixed senesced “community litter” were decomposed within a larger fertilization and grazing experiment (described in detail by [12]).The experiment is a split-plot design where fertilizer treatment is the whole-plot treatment and sheep grazing intensity were the sub-plot treatments. Four replicates were collected from each sub-plot after 1, 3, 6, 12, and 18 months. Litterbags were also collected from adjacent unfertilized and ungrazed control sites. Average percent of initial mass remaining at the end of the experiment was 40% (SE = 0.002). This experiment is described in detail in [13].

We used the Hobbie [14] data set for late stage or long-term decomposition. Average mass remaining at the final collection was 27.6% (SE = 0.60%). These data consisted of the data within [14] plus Hobbie’s unpublished filter paper mass loss data from the same experiment (hereafter, the Hobbie data set). Briefly, the Hobbie [14] experiment was established at Cedar Creek Ecosystem Science Reserve in central Minnesota, USA (45.40° N, 93.20° W; MAT = 6.7°C, MAP = 800 mm). Eight litters were decomposed for five years (1763 days) at eight sites (two old fields, a hardwood forest, two oak stands, two pine stands, and an aspen stand), with a nitrogen addition treatment at each site (6 replicates per treatment/time point). Details are presented in [14],[15].

**Figure 3:**
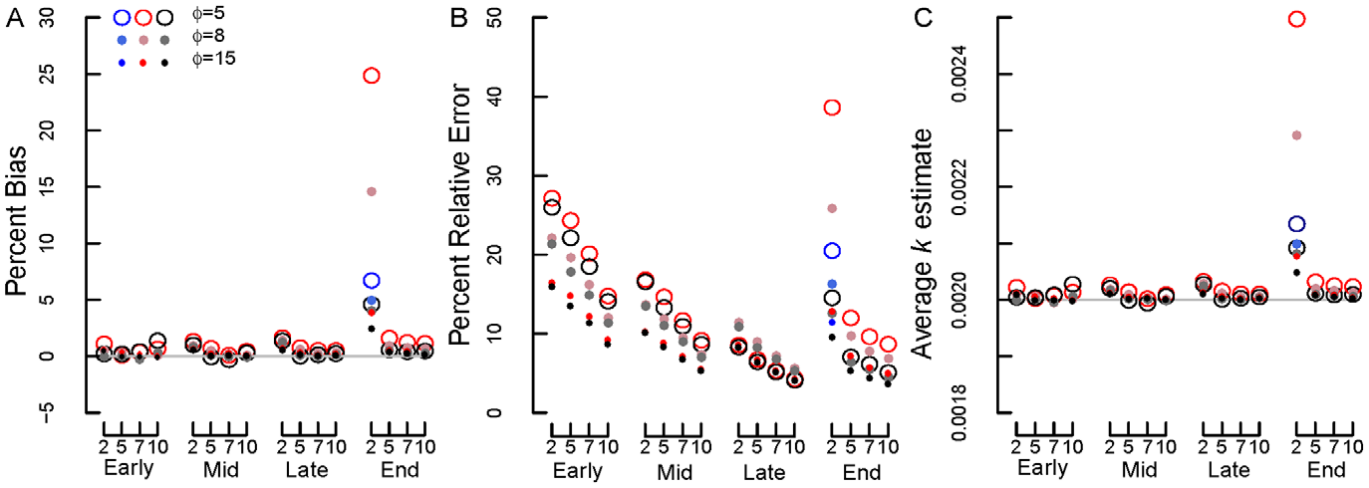
Simulation results for beta-distributed errors (option 2), *k* = 0.002. (A) Percent bias, (B) percent relative error, and (C) average *k* estimate. Early, mid, late and end are early, mid, late and end stage decomposition simulations. The numbers 2, 5, 7 and 10 are the numbers of measurements used in each simulation. Blue circles = NLS, Red circles = Normal ML, gray/black circles = Beta ML. In most cases, nls = Normal ML so that the red circles cover the blue circles. In panel (A), the gray line shows 0% bias. In panel (C), the gray line shows the true *k* value, 0.002 d^−1^.

**Figure 4:**
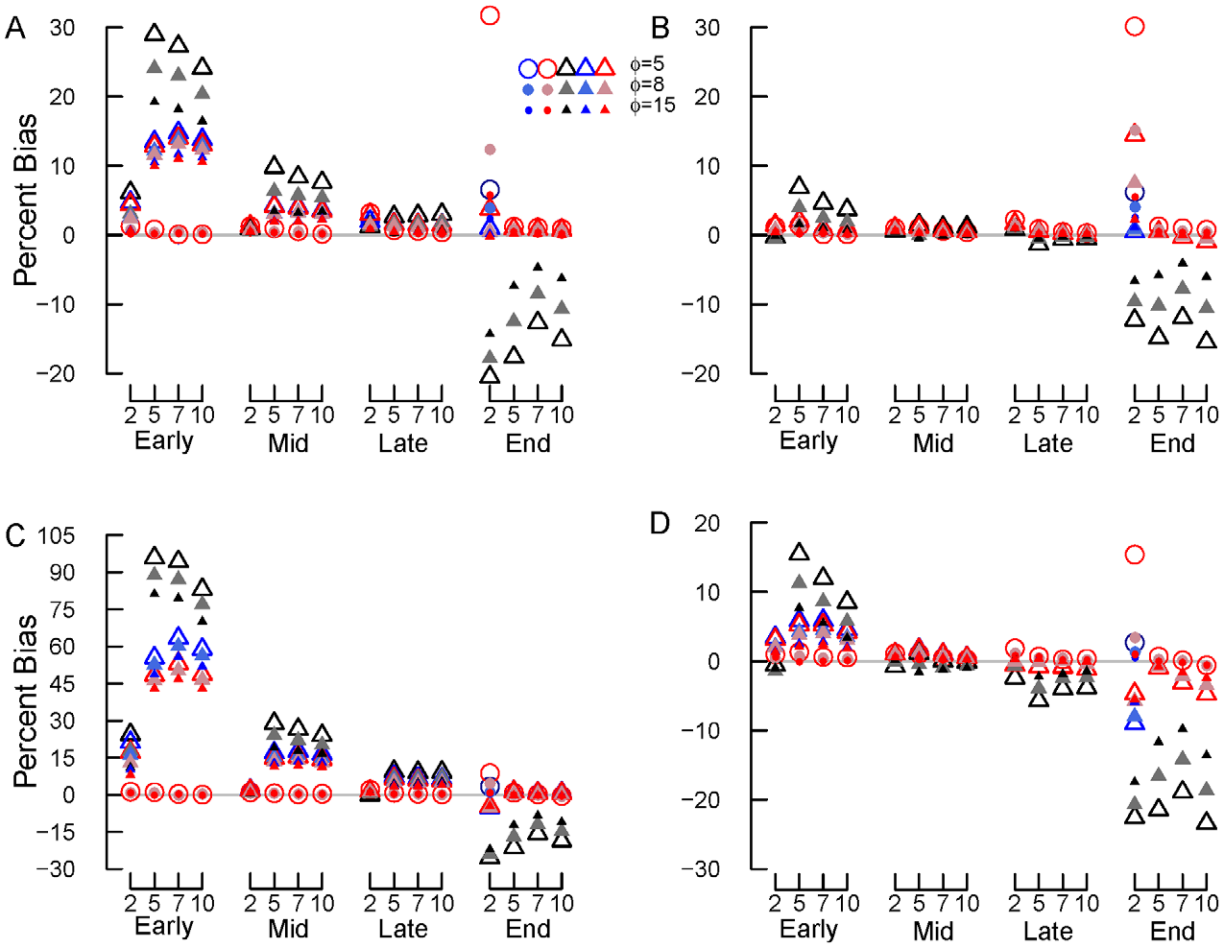
Percent bias for beta-distributed errors plus normal errors. (A) standard deviation (σ) = 0.0125 (option 3a) and SV transformation, (B) σ = 0.0125 (option 3a) and REP transformation, (C) σ = 0.05 (option 3b) and SV transformation, (D) σ = 0.05 (option 3b) and REP transformation. Early, mid, late and end are early, mid, late and end stage decomposition simulations. The numbers 2, 5, 7 and 10 are the numbers of measurements used in each simulation. Blue circles = NLS, Red circles = Normal ML, gray/black circles = Beta ML. In most cases, nls = Normal ML so that the red circles cover the blue circles. Gray lines show 0% bias.

**Figure 5:**
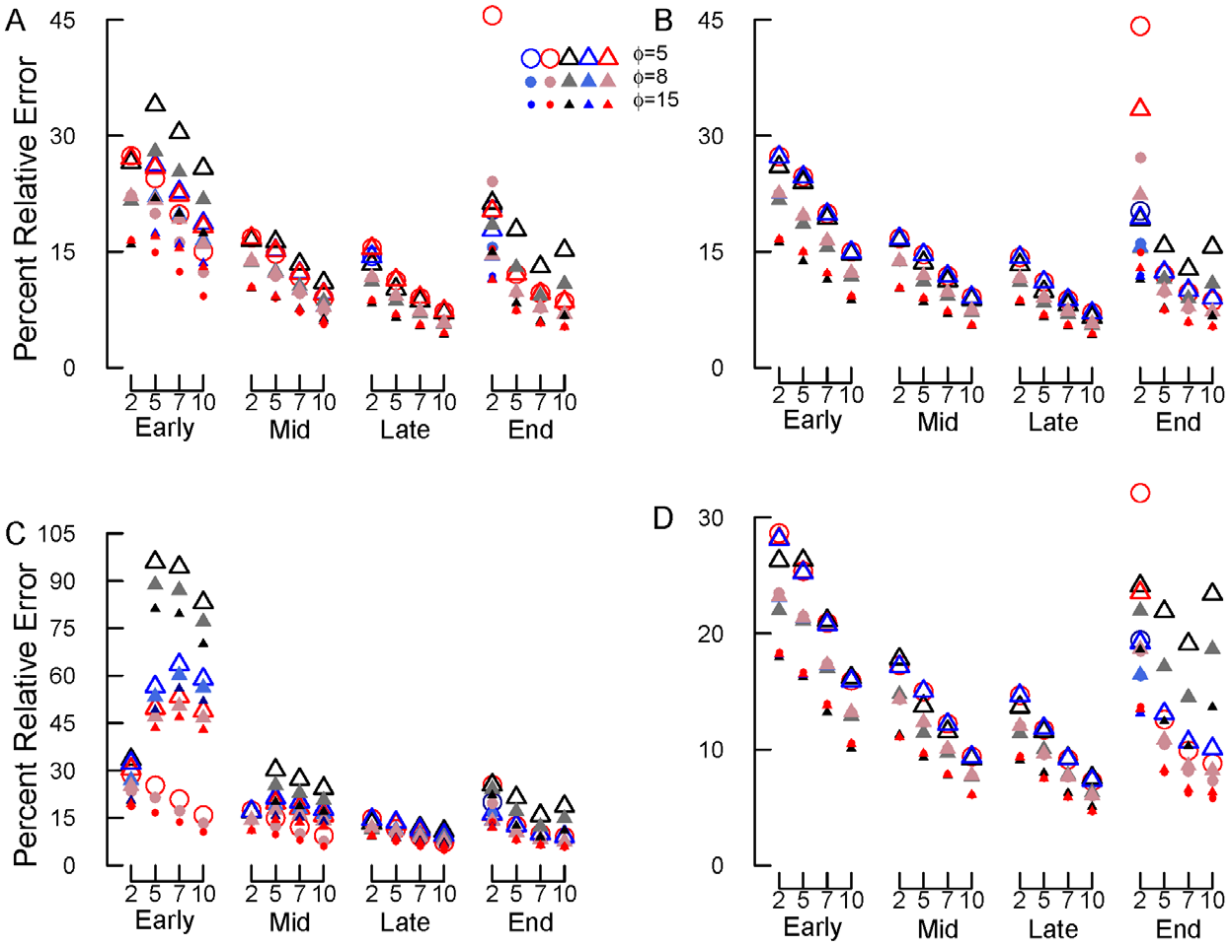
Percent relative error for beta-distributed errors plus normal errors with different σ and transformations. (A) σ = 0.0125 (option 3a) and SV transformation, (B) σ = 0.0125 (option 3a) and REP transformation, (C) σ = 0.05 (option 3b) and SV transformation, (D) σ = 0.05 (option 3b) and REP transformation. Early, mid, late and end are early, mid, late and end stage decomposition simulations. The numbers 2, 5, 7 and 10 are the numbers of measurements used in each simulation. Blue circles = NLS, Red circles = Normal ML, gray/black circles = Beta ML. In most cases, nls = Normal ML so that the red circles cover the blue circles.

**Figure 6:**
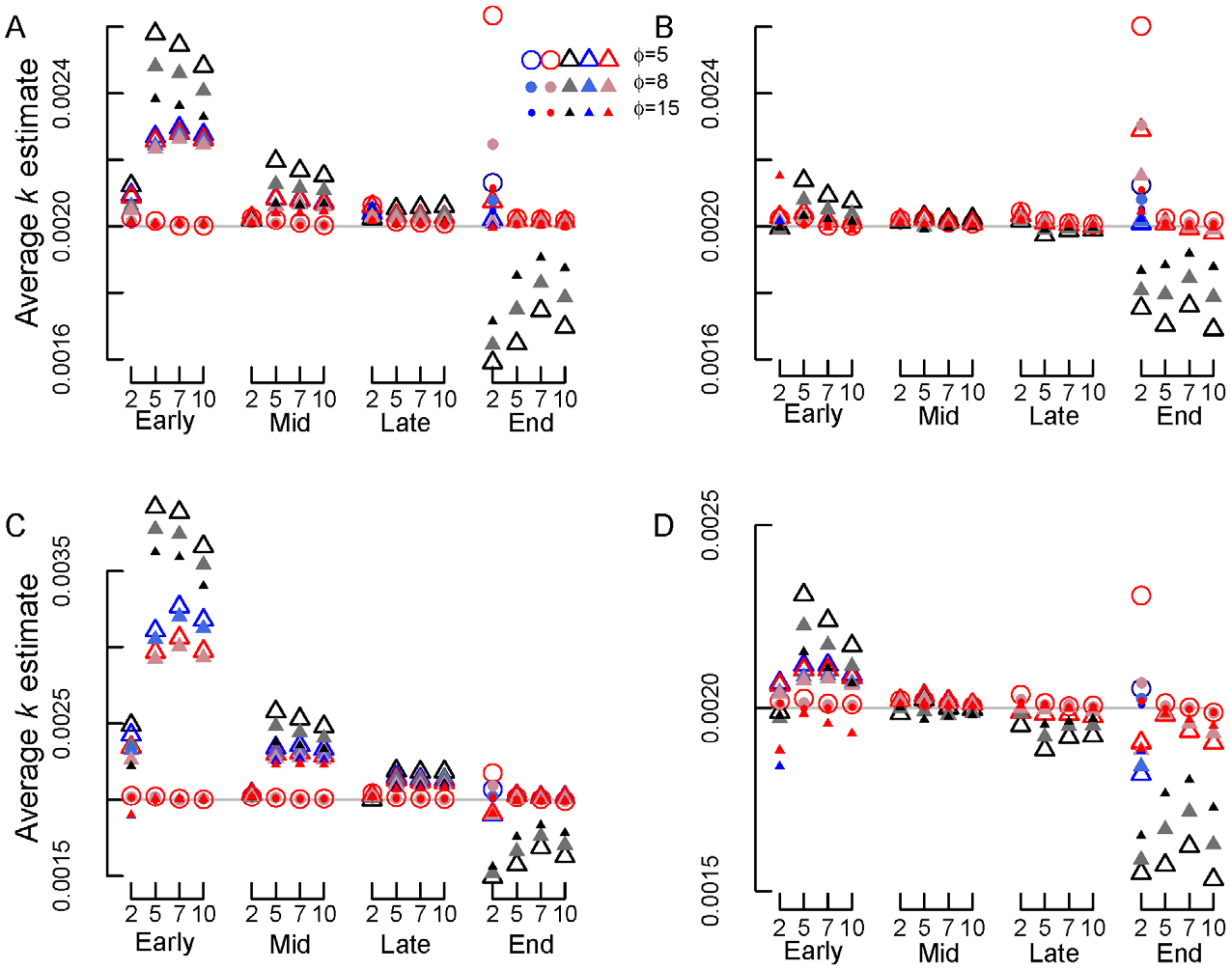
Average *k* estimates for beta-distributed errors plus normal errors with different σ and transformations. (A) σ = 0.0125 (option 3a) and SV transformation, (B) σ = 0.0125 (option 3a) and REP transformation, (C) σ = 0.05 (option 3b) and SV transformation, (D) σ = 0.05 (option 3b) and REP transformation. Early, mid, late and end are early, mid, late and end stage decomposition simulations. The numbers 2, 5, 7 and 10 are the numbers of measurements used in each simulation. Blue circles = NLS, Red circles = Normal ML, gray/black circles = Beta ML. In most cases, nls = Normal ML so that the red circles cover the blue circles. Gray lines show the true *k* value of 0.002 d^−1^.

**Figure 7:**
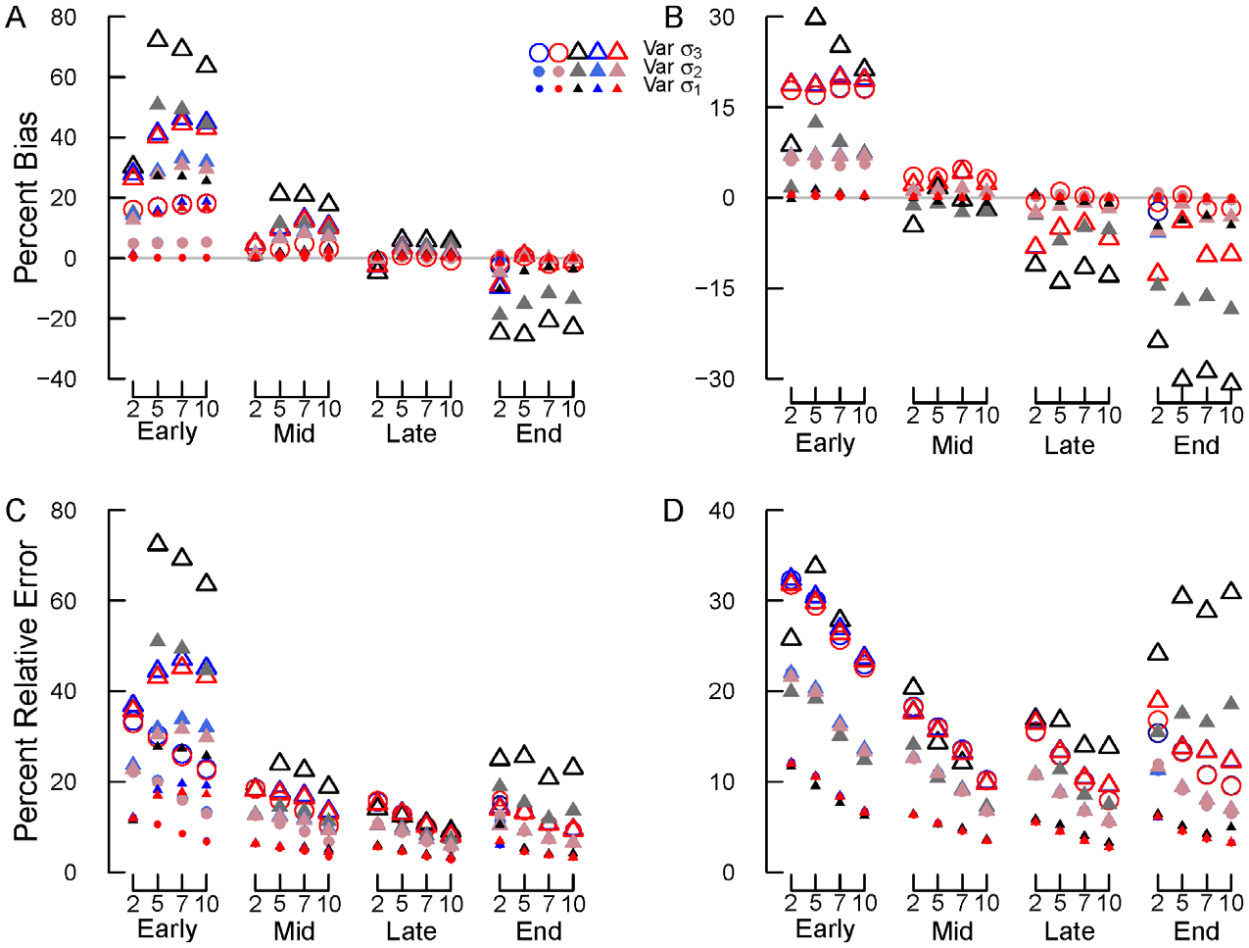
Results for simulations with variable normal errors (option 1). Percent bias using (A) SV and (B) REP transformations and relative error using (C) SV and (D) REP transformations. Early, mid, late and end are early, mid, late and end stage decomposition simulations. The numbers 2, 5, 7 and 10 are the numbers of measurements used in each simulation. Blue circles = NLS, Red circles = Normal ML, gray/black circles = Beta ML. In most cases, nls = Normal ML so that the red circles cover the blue circles. Gray lines in panels (A) and (B) show 0% bias.

**Figure 8:**
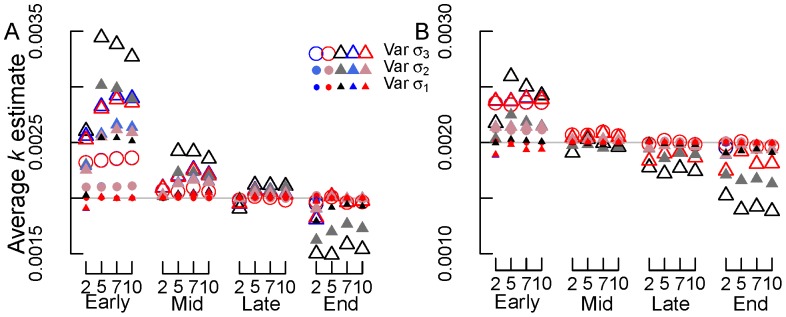
Average *k* estimates for simulations with variable normal errors (option 1). (A) SV and (B) REP transformations. Early, mid, late and end are early, mid, late and end stage decomposition simulations. The numbers 2, 5, 7 and 10 are the numbers of measurements used in each simulation. Blue circles = NLS, Red circles = Normal ML, gray/black circles = Beta ML. In most cases, nls = Normal ML so that the red circles cover the blue circles. Gray lines in panels (A) and (B) show the true *k* value of 0.002 d^−1^.

Because NLS and ML estimation using normal errors produced nearly identical results in the data simulations for early to late stage decomposition (Figures 3,4,5,6,7,8), we only compared *k* estimates obtained using ML estimation with normal and beta errors (*k* estimates/decomposition models were compared using AIC*c*; see below). As in the simulations, when using beta errors to estimate low *k* values (*k* <0.0015 d^−1^), optimization algorithms often failed to converge. In these cases, estimating *k* in years solved the problem. Thus, while all other *k* values were estimated as daily rates, *k* values in the Hobbie and Gough [10] data set were estimated as yearly rates and converted to daily rates for figures and tables.

We fit single pool models (Equation 2) to all litter mass loss curves within each data set using ML estimation with normal or beta errors (‘bbmle’ package version 1.0.4.1) [8]. The early, mid and late stage decomposition data sets contained 18, 64, and 128 litter mass loss curves, respectively. Whenever possible (i.e. all *X*(*t*) >0 and <1), we used untransformed data. When transformation was required, we used both the SV and REP data transformations. Within transformed or untransformed data sets, we compared model fit using AIC*_c_*. We considered models with AIC*_c_* between 4 and 7 apart (4< ΔAIC*_c_* <7) as clearly distinguishable and models with ΔAIC*_c_* >10 as definitely different, following previous recommendations [16].

We also examined model fit to the untransformed data using fractional bias (FB)
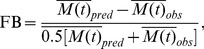
(22)and relative bias (RB)
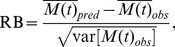
(23)where 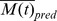
is the mean of predicted values, 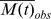 is the mean of all observations, and var[M(t)obs] is the sample variance of all observations [17]. These metrics express the average amount of bias in the model predictions (compared to the observations) and thus describe the ‘model-data’ discrepancy [17].

## Results

### Simulations

#### Beta-distributed errors

For beta-distributed errors (option 2; no data transformations needed), the accuracy of *k* estimates generally increased with the number of measurements (from two to ten) and declining error (from *φ* = 5 to 15; Figure 3). Bias and RE decreased with increasing number of measurements and with decreasing error (*φ*). In general, *k* estimates also improved (reduced bias and RE) from early to late decomposition (Figure 3). However, when study lengths were the longest (end stage decomposition), using only two measurements often resulted in inaccurate *k* estimates, particularly when using ML estimation with normal errors.

Across all simulations, using ML regression with beta errors resulted in very similar or more accurate *k* estimates than NLS or ML with normal errors (Figure 3). This was particularly true for end stage decomposition with two measurements, where the beta model provided more accurate *k* estimates than the normal models (i.e., lower bias and RE, average *k* closer to true *k* of 0.002; Figure 3). However, beta ML regression did not successfully converge for all the data sets produced by the simulations (Table 2). Beta ML regression most often failed to converge during early decomposition, when *φ* <15, and the number of measurements was >2 (Table 2). In contrast, both NLS and normal ML estimation consistently successfully estimated *k*. In general, NLS and normal ML estimation produced nearly identical results. The exception was end stage decomposition with two measurements – in this case NLS produced slightly more accurate *k* estimates than did ML estimation with normal errors.

In most cases, AIC*_c_* identified ML estimation with beta errors as the best model (Table 3). In the majority of simulations, ML estimation with beta errors was identified as the best model in 90–100% of cases (Table 3). In the remaining simulations, AIC*_c_* generally showed either no difference between ML estimation with beta and normal errors (13.3–99.2% of cases) or found ML estimation with beta errors to be the best model (0–86.5% of cases; Table 3). Across all simulations, ML estimation with normal errors was only identified as the best model in 0–3% of cases.

**Table 3:** Percent of simulations using beta errors (option 2) for which AIC*c* selected maximum likelihood (ML) estimation with beta or normal errors best or found no difference between the two models (Same) from each simulation (*k = *0.0002).

		*φ* = 5			*φ* = 8			*φ* = 15		
Stage	# meas^2^	Same	Beta ML	Norm ML	Same	Beta ML	Norm ML	Same	Beta ML	Norm ML
Early	2	35.3	64.3	0.4	66.5	32.5	1.0	91.7	6.0	2.3
	5	0.0	100.0	0.0	0.0	100.0	0.0	0.1	99.9	0.0
	7	0.0	100.0	0.0	0.0	100.0	0.0	0.0	100.0	0.0
	10	0.0	100.0	0.0	0.0	100.0	0.0	0.0	100.0	0.0
Mid	2	98.3	1.2	0.6	99.2	0.0	0.8	99.2	0.0	0.8
	5	0.2	99.8	0.0	1.3	98.7	0.0	8.4	91.3	0.3
	7	0.1	99.9	0.0	0.6	99.4	0.0	4.2	95.6	0.2
	10	0.0	100.0	0.0	0.1	99.9	0.0	1.1	98.9	0.1
Late	2	87.6	11.6	0.8	97.0	1.6	1.4	97.8	0.0	2.2
	5	6.3	93.6	0.1	24.3	75.3	0.4	62.5	35.8	1.8
	7	3.4	96.6	0.1	13.3	86.5	0.2	40.7	58.2	1.2
	10	0.4	99.6	0.0	3.8	96.2	0.1	18.6	80.9	0.5
End	2	0.0	100.0	0.0	0.2	99.8	0.0	1.0	98.9	0.1
	5	0.0	100.0	0.0	0.0	100.0	0.0	0.1	99.9	0.0
	7	0.0	100.0	0.0	0.0	100.0	0.0	0.1	99.9	0.0
	10	0.0	100.0	0.0	0.0	100.0	0.0	0.0	100.0	0.0

Results were similar across all *k* values.

1 Norm = normal.

2 meas = measurements.

#### Beta-distribution errors with normal errors added

For simulations with beta-distributed plus normal errors (option 3), the accuracy of *k* estimates again tended to increase (i.e., bias and RE declined) with the number of measurements and declining error (from *φ* = 5 to 15 and normal error σ = 0.05 to 0.0125; Figures 4,5,6). Estimates of *k* also improved from early to late stage decomposition (Figures 4,5,6). However, during end stage decomposition, *k* estimates became more variable, particularly when *k* was estimated using only two measurements (Figures 4,5,6).

With few exceptions, estimating *k* using NLS and ML estimation with normal errors on untransformed data produced similar (to one another) and more accurate *k* estimates (lower bias and RE) than did transforming the data and using NLS or ML estimation with normal or beta errors (Figures 4,5,6). The only exception was for the end stage decomposition simulation with only two measurements, where using ML estimation with normal errors produced high bias and RE (Figures 4–5). In general, beta regression on transformed data resulted in high bias and RE (Figures 4,5,6). These differences were most apparent in the early and end stage decomposition simulations; the smallest amount of bias and RE among estimation and transformation techniques (and thus *k* estimates) occurred during mid and late stage decomposition.

Overall, the REP transformation resulted in less bias and RE than did the SV transformation. This was especially apparent in early, mid and late stage decomposition. The amount of bias and RE generated by the REP and SV transformations was similar during end stage decomposition.

Despite the fact that ML estimation using beta errors tended to generate less accurate *k* estimates than ML estimation using normal errors, AIC*_c_* generally showed either no difference between ML estimation using beta and normal errors or found ML estimation with beta errors to be the best model. For SV and REP transformed data with low normal error (σ = 0.0125), AIC*_c_* either selected ML estimation with beta errors as the best model or found no difference between ML selection with beta and normal errors. Only during end stage decomposition with two measurements was ML estimation using normal errors selected as the best model more than 3% of the time (Table 4).

**Table 4:** Percent of beta error simulations with normal error (σ = 0.0125) added, for which AIC*c* selected maximum likelihood (ML) estimation with beta or normal errors best or found no difference between the models (Same) from each simulation (*k = *0.0002).

			*φ* = 5			*φ* = 8			*φ* = 15		
Tr^1^	Stage	# meas	Same	Beta ML	Norm ML	Same	Beta ML	Norm ML	Same	Beta ML	Norm ML
SV^2^	Early	2	50.6	49.0	0.4	74.6	24.4	0.9	91.6	5.7	2.7
		5	4.6	94.6	0.8	8.7	90.3	1.0	16.4	81.5	2.0
		7	1.1	98.9	0.1	3.3	96.6	0.2	10.4	88.5	1.1
		10	0.3	99.7	0.0	1.5	98.4	0.1	6.1	93.0	0.9
	Mid	2	98.0	1.2	0.8	98.9	0.0	1.1	99.1	0.0	0.9
		5	2.3	97.6	0.1	6.2	93.7	0.1	16.8	82.7	0.5
		7	1.0	99.0	0.0	3.5	96.4	0.1	10.9	88.6	0.5
		10	0.5	99.5	0.0	1.6	98.4	0.1	5.5	94.2	0.3
	Late	2	89.2	10.1	0.7	96.9	1.5	1.6	97.8	0.0	2.2
		5	9.5	90.4	0.1	28.8	70.8	0.5	62.7	35.4	1.9
		7	5.4	94.5	0.1	17.1	82.5	0.4	43.9	55.0	1.1
		10	1.0	99.0	0.0	5.9	94.0	0.1	21.4	77.7	0.9
	End	2	34.3	43.6	22.1	41.8	35.5	22.6	49.0	25.6	25.5
		5	1.9	97.9	0.2	3.1	96.6	0.3	6.3	93.3	0.4
		7	0.1	99.9	0.0	0.6	99.4	0.0	2.1	97.8	0.1
		10	0.0	100.0	0.0	0.0	100.0	0.0	0.0	100.0	0.0
REP^3^	Early	2	32.8	66.9	0.4	62.2	36.5	1.3	89.2	7.4	3.5
		5	0.0	100.0	0.0	0.0	100.0	0.0	0.4	99.5	0.1
		7	0.0	100.0	0.0	0.0	100.0	0.0	0.2	99.8	0.0
		10	0.0	100.0	0.0	0.0	100.0	0.0	0.1	99.9	0.0
	Mid	2	97.7	1.6	0.8	99.2	0.1	0.8	99.1	0.0	0.9
		5	0.4	99.6	0.0	1.7	98.2	0.1	9.4	89.8	0.8
		7	0.2	99.8	0.0	1.2	98.8	0.1	5.5	94.0	0.5
		10	0.0	100.0	0.0	0.5	99.5	0.0	1.9	97.9	0.2
	Late	2	90.6	8.7	0.8	97.1	1.5	1.4	97.6	0.0	2.4
		5	6.9	93.0	0.2	24.0	75.3	0.7	62.3	34.8	2.9
		7	3.9	96.0	0.1	13.6	85.9	0.5	43.4	54.9	1.7
		10	0.7	99.3	0.0	4.4	95.5	0.2	19.5	79.2	1.4
	End	2	39.1	53.3	7.7	44.9	46.3	8.9	53.1	36.3	10.6
		5	8.2	91.1	0.8	11.0	88.2	0.8	14.5	84.4	1.1
		7	1.2	98.8	0.0	3.1	96.7	0.1	7.2	92.4	0.4
		10	0.1	100.0	0.0	0.2	99.8	0.0	1.1	98.8	0.2

1 Tr = transformation.

2 SV = Smithson and Verkuilen [6] transformation.

3 REP = transformed by replacing values ≥1 with 0.9999 and treating zeros as missing data.

In SV transformed data with high normal error (σ = 0.05), AIC*_c_* more frequently selected ML estimation with normal errors as the best model, particularly in early decomposition simulations with more than two measurements and end stage decomposition simulations with only two measurements (Table 5). In REP transformed data with high normal error (σ = 0.05), AIC*_c_* again found either no difference between models or ML estimation with beta errors as the best model in the majority of cases across all simulations. Only during end stage decomposition was ML estimation with normal errors selected as the best model more than 8% of the time.

**Table 5:** Percent of beta error simulations with normal error (σ = 0.05) added, for which AIC*c* selected maximum likelihood (ML) estimation with beta or normal errors best or found no difference between the models (Same) from each simulation (*k = *0.0002).

			*φ* = 5			*φ* = 8			*φ* = 15		
Tr^1^	Stage	# meas	Same	Beta ML	Norm ML	Same	Beta ML	Norm ML	Same	Beta ML	Norm ML
SV^2^	Early	2	73.7	25.9	0.4	85.1	13.9	1.0	92.8	4.3	2.9
		5	33.7	19.3	46.9	31.7	13.2	55.1	25.5	6.9	67.6
		7	36.3	27.8	35.8	33.4	17.6	49.0	26.9	8.6	64.5
		10	31.4	25.9	42.7	26.9	14.6	58.4	17.3	6.2	76.6
	Mid	2	97.8	1.3	0.9	98.4	0.2	1.5	98.2	0.0	1.9
		5	40.1	55.1	4.9	46.9	44.9	8.2	53.1	31.8	15.2
		7	34.2	60.9	4.9	43.1	48.6	8.4	50.0	33.8	16.2
		10	33.9	58.7	7.3	40.2	46.8	13.0	43.6	33.9	22.5
	Late	2	90.5	8.7	0.9	96.7	1.9	1.4	96.3	0.2	3.5
		5	26.4	73.2	0.4	46.8	51.1	2.1	71.4	22.4	6.2
		7	20.3	79.0	0.8	38.8	58.8	2.4	60.4	32.9	6.7
		10	9.1	90.5	0.4	25.1	72.5	2.4	47.8	43.8	8.4
	End	2	41.5	20.8	37.8	45.6	13.7	40.7	44.4	6.2	49.4
		5	12.2	85.4	2.4	18.0	78.6	3.4	28.7	66.0	5.3
		7	1.9	97.9	0.3	6.1	93.2	0.7	15.5	81.8	2.7
		10	0.1	99.9	0.0	0.5	99.4	0.1	2.8	96.7	0.5
REP^3^	Early	2	27.1	72.1	0.8	47.2	50.7	2.1	72.5	21.8	5.7
		5	0.1	99.8	0.0	0.4	99.5	0.1	1.2	98.4	0.4
		7	0.0	100.0	0.0	0.1	99.9	0.0	0.9	98.9	0.3
		10	0.0	100.0	0.0	0.0	100.0	0.0	0.4	99.5	0.1
	Mid	2	93.3	5.7	1.0	97.2	0.8	2.0	97.6	0.0	2.4
		5	0.8	99.1	0.1	3.2	96.5	0.3	10.6	87.3	2.1
		7	0.6	99.4	0.1	2.7	96.8	0.5	9.5	87.5	3.0
		10	0.2	99.7	0.1	1.8	97.8	0.5	5.9	91.9	2.3
	Late	2	93.1	5.8	1.0	97.1	1.4	1.6	96.4	0.1	3.5
		5	9.1	90.4	0.5	24.1	73.7	2.2	54.3	38.7	7.1
		7	6.6	92.7	0.8	15.8	82.1	2.2	39.4	54.8	5.8
		10	2.0	97.8	0.2	7.0	91.9	1.1	23.4	71.7	4.9
	End	2	61.9	17.0	21.1	63.1	12.9	24.0	61.3	7.5	31.2
		5	36.1	56.0	8.0	46.3	41.2	12.5	53.5	28.3	18.2
		7	16.2	81.7	2.1	30.2	64.7	5.1	42.7	44.6	12.7
		10	6.0	93.1	0.9	14.1	82.6	3.3	28.6	59.3	12.1

1 Tr = transformation.

2 SV = Smithson and Verkuilen [6] transformation.

3 REP = transformed by replacing values ≥1 with 0.9999 and treating zeros as missing data.

#### Variable σ Normal Error

Again, percent bias and RE declined from early to late stage decomposition and RE declined with number of measurements (Figures 7–8). Estimates of *k* also improved with declining error (from Var σ_1_ to Var σ_3_; Figures 7–8). However, increasing the number of measurements within decomposition stage failed to reduce percent bias and did not typically improve average *k* estimates (Figures 7–8). Again, bias and RE increased during end stage decomposition (Figures 7–8), relative to mid and late stage decomposition simulations.

Using NLS or ML estimation with normal errors on untransformed data yielded the most consistently accurate *k* values with low bias and relative error across all decomposition stages and numbers of measurements (Figures 7–8). For transformed data, using the SV or REP transformation combined with NLS or ML estimation with normal errors frequently resulted in less bias and relative error than using ML estimation with beta errors (Figure 7). In certain cases, using beta regression on transformed data resulted in *k* values that were just as or more accurate than other methods: most frequently this occurred during mid and late stage decomposition.

In general, using the REP transformation resulted in less bias and relative error than did using the SV transformation (Figure 7). This was especially true during early to late stage decomposition. During end stage decomposition, both transformations generated similar levels of bias and relative error (Figure 7).

When the data were SV transformed, across all decomposition stages, numbers of measurements, and amounts of error used to create the simulated data, AICc generally identified ML estimation with beta errors as the best model or found no difference between ML estimation with beta or normal errors (Table 6). However, ML estimation with normal errors was identified as the best model more frequently than when other error structures were used to generate the data (i.e., beta or beta plus normal errors). In particular, AIC*_c_* identified ML estimation with normal errors as the best model more frequently during early decomposition with more than two measurements, in end stage decomposition with only two measurements, and in mid and late decomposition when error was low (Var σ_1_ and Var σ_2_).

**Table 6:** Percent of variable normal σ simulations for which AIC*c* selected maximum likelihood (ML) estimation with beta or normal errors best or found no difference between the models (Same) from each simulation (*k = *0.0002).

			Var σ_3_			Var σ_2_			Var σ_1_		
Tr^1^	Stage	# meas	Same	Beta ML	Norm ML	Same	Beta ML	Norm ML	Same	Beta ML	Norm ML
SV^2^	Early	2	75.1	24.1	0.8	90.1	7.1	2.8	90.7	0.1	9.2
		5	39.3	32.5	28.1	39.3	21.7	39.0	39.7	22.6	37.8
		7	37.3	44.2	18.5	39.3	21.1	39.6	35.5	16.7	47.8
		10	31.8	51.3	16.9	33.1	20.7	46.2	26.8	14.9	58.3
	Mid	2	95.8	3.0	1.3	96.5	0.1	3.5	98.3	0.0	1.7
		5	36.7	57.3	6.0	45.7	47.7	6.6	48.1	41.4	10.5
		7	29.4	66.6	3.9	44.6	43.6	11.8	43.7	38.9	17.4
		10	30.7	60.1	9.3	39.9	41.7	18.5	37.1	47.9	15.0
	Late	2	91.5	7.6	0.9	95.8	0.9	3.3	89.5	0.0	10.5
		5	30.5	67.4	2.1	63.6	27.8	8.6	74.7	1.3	24.0
		7	28.1	68.8	3.0	56.5	31.4	12.2	68.4	3.5	28.1
		10	14.0	84.2	1.8	46.1	39.1	14.8	55.0	6.1	38.9
	End	2	54.5	8.3	37.2	49.1	5.2	45.8	56.7	5.8	37.5
		5	21.4	73.0	5.6	24.2	70.8	5.0	23.0	74.8	2.1
		7	6.2	92.9	0.9	14.6	82.7	2.7	20.0	75.5	4.5
		10	0.6	99.3	0.1	1.9	97.7	0.4	2.3	97.3	0.4
REP^3^	Early	2	23.2	74.8	2.0	53.1	39.6	7.3	85.6	0.5	14.0
		5	0.6	99.2	0.2	1.9	97.4	0.7	10.3	86.3	3.4
		7	0.1	99.8	0.0	1.2	98.3	0.6	13.2	79.6	7.2
		10	0.0	100.0	0.0	0.4	99.4	0.1	8.8	85.0	6.2
	Mid	2	77.9	19.3	2.8	92.7	1.1	6.3	98.4	0.0	1.7
		5	3.9	95.1	1.0	13.9	82.7	3.5	42.4	37.7	19.9
		7	1.5	98.1	0.4	10.6	84.8	4.5	35.2	42.3	22.6
		10	1.3	98.2	0.5	8.6	86.4	5.0	26.2	55.3	18.6
	Late	2	95.6	2.9	1.5	95.9	0.3	3.8	90.2	0.0	9.9
		5	11.7	86.0	2.4	39.4	49.4	11.2	66.2	1.3	32.5
		7	10.3	86.4	3.3	32.3	56.1	11.7	55.0	3.5	41.6
		10	6.0	91.8	2.2	21.3	69.5	9.3	42.4	4.9	52.8
	End	2	70.4	6.6	23.0	65.4	7.1	27.6	67.7	12.1	20.2
		5	37.3	48.7	14.0	48.7	35.4	16.0	35.5	60.3	4.3
		7	26.2	68.1	5.8	39.0	49.5	11.6	30.9	60.8	8.4
		10	9.2	88.4	2.4	16.5	78.3	5.2	7.4	86.7	5.9

1 Tr = transformation.

2 SV = Smithson and Verkuilen [6] transformation.

3 REP = transformed by replacing values ≥1 with 0.9999 and treating zeros as missing data.

When using the REP transformation, AIC*_c_* usually selected ML estimation with beta errors as the best model or found no difference between the models, especially when error was high or moderate (Var σ_2_ and Var σ_3_) and the number of measurements was more than two (Table 6). When error was low (Var σ_1_), AIC*_c_* more frequently showed ML estimation with normal errors to be the best model.

### Real Data

#### Early stage decomposition data (hobbie and gough [10])

**Figure 9:**
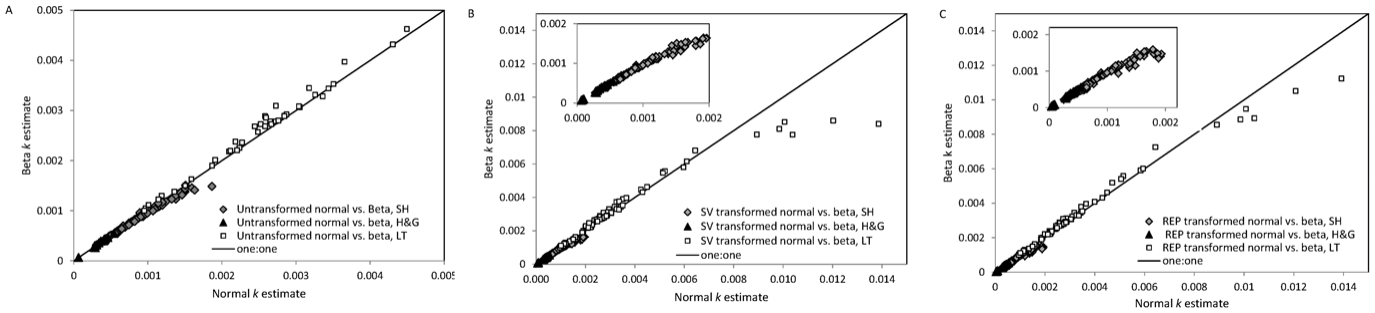
Daily decomposition rate (*k*) estimates for the Hobbie (SH) [**14**], Laliberté and Tylianakis (LT) [**12**] and Hobbie and Gough (H&G) [**10**] data compared by error distribution (beta or normal) used to estimate *k_._* (A) untransformed (B) Smithson and Verkuilen (SV) [**6**] transformed and (C) replacement (zeros = missing data; values ≥1 = 0.9999) transformed data sets. Insets in (b) and (c) show only the SH and H&G data.

Overall, normal and beta errors produced similar *k* estimates within the transformed and untransformed data sets (Figure 9, Table 7). Fractional and relative bias for all transformation and error combinations were relatively small, but the SV transformation resulted in either similar or slightly more bias than the REP transformation or no transformation (Table 7). Within the untransformed and REP transformed data sets, using beta errors produced less bias than using normal errors.

**Table 7:** Mean *k* (decomposition rate), fractional bias (FB) and relative bias (RB) produced by each data transformation and error structure using the Hobbie [14], Laliberté and Tylianakis [12] and Hobbie and Gough [10] data sets.

Data	Transformation	Error	Mean *k*(d^−1^)	Mean FB	σ FB	Mean RB	σ RB
Hobbie &Gough							
	None	Beta	0.00055	0.0001	0.0020	0.0047	0.0190
		Normal	0.00054	0.0026	0.0088	0.0308	0.1163
	SV^1^	Beta	0.00055	−0.0042	0.0083	−0.1394	0.2908
		Normal	0.00054	−0.0006	0.0121	−0.0758	0.2923
	REP^2^	Beta	0.00055	−0.0002	0.0018	−0.0049	0.0276
		Normal	0.00054	0.0024	0.0089	0.0263	0.1216
Laliberté &Tylianakis							
	None	Beta	0.00258	0.1090	0.0481	0.2778	0.0985
		Normal	0.00363	0.1682	0.1082	0.3399	0.1219
	SV^1^	Beta	0.00349	0.1646	0.1438	0.3256	0.1979
		Normal	0.00361	0.1693	0.1098	0.3416	0.1244
	REP^2^	Beta	0.00357	0.0192	0.0449	0.0279	0.0647
		Normal	0.00356	0.0286	0.0303	0.0561	0.0495
Hobbie							
	None	Beta	0.00088	0.0123	0.0293	0.0249	0.0660
		Normal	0.00091	−0.0142	0.0227	−0.0236	0.0574
	SV^1^	Beta	0.00090	−0.0097	0.0604	−0.0291	0.1247
		Normal	0.00095	−0.0343	0.0562	−0.0636	0.1192
	REP^2^	Beta	0.00084	0.0220	0.0453	0.0330	0.0675
		Normal	0.00091	−0.0148	0.0232	−0.0251	0.0588

1 SV = Smithson and Verkuilen [6] transformation.

2 REP = data transformed by replacing values ≥1 with 0.9999 and treating zeros as missing data.

In 13 of 18 cases, the beta distribution could be used on untransformed data (all values >0 and <1). In these cases the beta model was best (ΔAIC*_c_* ≥4) in four cases. In the nine remaining cases, the models were indistinguishable based on AIC*_c_*. When the data were SV transformed, the beta distribution produced the best model in five cases, but in the remaining 13 cases the models were indistinguishable. For REP transformed data, the beta model was best in nine cases; for the remaining nine cases the models were indistinguishable.

#### Mid stage decomposition data (laliberté and tylianakis [12])

Using normal and beta errors generally produced very similar *k* estimates (Figure 9). Notable exceptions were when the data were transformed and *k* was greater than ∼0.01 d^−1^ (Figure 9b,d), in which case the normal model gave larger *k* estimates than the beta model (Figure 9b,d). This was particularly evident when using the SV transformation (Figure 9b). In these cases, the beta model produced more biased predictions than the normal model (Table S1). For the SV transformed data, FB and RB were 14–60% larger for the beta than normal model. For the REP transformed data, FB and RB were 1.4 to 18 times larger for the beta than normal model (Table S1). Despite the larger bias associated with the beta model for the SV data, the beta model was identified as best in three cases (ΔAIC*_c_* ≥4; Table S1). In the remaining cases the models were indistinguishable (ΔAIC*_c_* <4; Table S1). For the REP transformed data, the models were indistinguishable in all cases (Table S1).

In general, using the SV data transformation resulted in similar or slightly more bias than the REP or no transformation (Table 7). Within the untransformed and REP transformed data, using beta errors produced predictions with similar or less bias than did using normal errors.

In 40 of 64 cases, the data did not need to be transformed to use beta errors. Based on AIC*_c_*, the beta model was best (ΔAIC*_c_* ≥4) in only three of these cases. In 18 cases the normal model was best. For the remaining cases, the models were indistinguishable. With the SV transformed data, the beta model was best in 14 cases, while the normal model was best in 18 cases. Using the REP transformation, the beta model was best in four cases, while the normal model was best in 18 cases. The models were indistinguishable in all remaining cases.

#### Late stage decomposition data (hobbie [14])

Again, normal and beta distributed errors produced largely similar *k* estimates within the same data set (Figure 9). However, at high *k* values (>0.0015 d^−1^), beta models produced slightly lower *k* estimates than normal models (Fig 9b,c). Unlike medium stage data, this was also true for untransformed data, and there were no consistent patterns in bias for these points (Table S1). Of the 21 cases where *k* estimated using normal errors was ≥0.0015 d^−1^, for both the SV and REP transformations, the beta model was best in 11 cases, the normal model was best in nine cases, and there was no difference between the models in one case (Table S1). For the untransformed data, when *k* could be estimated using beta errors (9 of 21 cases), using the normal model resulted in less bias and was the best model (data not shown).

Using the SV transformation resulted in predictions with similar or more bias than using no transformation or the REP transformation (Table 7). The only exception was for the beta model, where less bias was generated using the SV than REP transformation. In the untransformed data set, normal and beta errors produced similar bias; in the REP transformed data, using beta errors produced slightly less bias than using normal errors (Table 7).

Of the 79 cases where the beta model could be used on untransformed data, it was best in 33, whereas the normal model was best in 22 cases. The models were indistinguishable in 24 cases. Using SV transformation, in 25 out of 128 cases there was no substantial difference between the models. In the majority of cases (69) the beta model was best. The normal model was best in 34 cases. Using the REP transformation, the beta model was best in 75 cases, the normal model was best in 29 cases, and the models were indistinguishable in 24 cases.

## Discussion

Proportional litter mass loss data generally show reduced variance near its bounds (i.e. 0 and 1), but researchers generally use single pool decomposition models that ignore such heteroscedasticity, potentially leading to biased *k* estimates [3]. For example, the most recent recommendation to use standard nonlinear regression on untransformed proportional mass loss data still assumes constant, normally-distributed errors (a problem acknowledged by these authors [3]). We therefore evaluated the potential of beta regression, which is well suited to bounded data and its associated heteroscedasticity [6],[7]. We hypothesized that nonlinear beta regression would provide a better fit to proportional litter mass loss data, and more accurate *k* estimates, than standard nonlinear regression in simulated and real decomposition data sets.

Contrary to our hypothesis, we found that standard nonlinear regression with constant, normal errors proved very robust to violations of homoscedasticity. In our simulations, *k* estimates obtained via the normal model (NLS or ML estimation) on untransformed data were equally or more accurate as those obtained with the beta model, regardless of error structure and data transformation. On transformed and untransformed (beta errors only) data, ML estimation using beta errors tended to generate less accurate *k* estimates than ML estimation using normal errors. This occurred despite the beta model being clearly equal or superior in nearly all cases to the normal model, as determined by AIC*_c_*. Thus, our concern that standard nonlinear regression may lead to biased *k* estimates in the presence of heteroscedasticity appears to be unjustified by our simulation results. However, we do not imply that researchers should use standard nonlinear regression even when its assumptions are violated, simply because these results did not show systematic biases in *k* estimates. Still, it is important to note that *k* values previously estimated in the presence of heteroscedasticity using standard nonlinear regression should not be strongly biased.

Our simulations also provided information for the design of decomposition experiments, suggesting that the accuracy of *k* estimates increases with the number of measurements and with the length of the study, from early to late decomposition. Estimates of *k* from end stage decomposition were less accurate (or at least more variable between estimation methods), perhaps due to an increasing number of zero measurements or missing data (REP transformation), which may bias estimates [3]. In general, mid and late stage decomposition had the least amount of between method and data transformation variation in *k* estimates, suggesting that studies in these ranges will be less impacted by regression method choice.

Obviously, with real proportional litter mass loss data we cannot evaluate how “biased” *k* estimates are, because we do not know the “true” *k* value (which is why it must be estimated from data). Yet, we must make an informed decision on which model provides the best estimate of *k*. Tools at our disposal include various measures of model fit such as AIC [18], and visual inspection of model predictions and residuals to evaluate model assumptions [19]. In untransformed or REP transformed real data, the beta model produced slightly less bias than did the normal model. Using AIC*_c_*, we found that the models were indistinguishable from each other in the majority of cases. Therefore, we recommend a pragmatic approach where both models are compared and the best one is selected for a given data set (particularly when *k* estimates are high and normal and beta model *k* estimations diverge). Alternatively, one may use model averaging to calculate the weighted average of *k* using both the beta and normal models [18]. This technique has been successfully used to estimate accurate parameters for biological power functions, where similar error structure issues are encountered (normal vs. lognormal models/errors) [5].

While we have focused on the beta distribution because it suits bounded data especially well [6],[7], several other distributions could be used to suit particular situations [16]. Yet, the beta distribution will be especially useful to estimate decomposition rates in single pool models because it easily accommodates the type of heteroscedasticity encountered in proportional mass loss data. In practice, a particular statistical model is often favored by researchers not just because it fits the data better, but for other pragmatic reasons such as computational simplicity [16],[19]. Unlike standard nonlinear regression, nonlinear beta regression is not widely implemented in mainstream statistical packages. This does not mean, however, that nonlinear beta regression is more complex than standard nonlinear regression with normal errors. Like the normal distribution, the beta distribution contains only two parameters and can be easily parameterized with location and precision (the inverse of dispersion) parameters [6], [7] (see Introduction). To facilitate the use of nonlinear beta regression in single pool decomposition models, we provide code to implement this approach in the freely available R environment [9] (Appendix S1). Because the beta distribution does not allow values ≤0 or ≥1, which often occur in proportional litter mass loss data, transformations to constrain the data in the ]0, 1[interval may be required. We evaluated two such transformations: the SV [6] and REP transformations. The SV transformation simultaneously standardizes all values, so the transformed data stay perfectly correlated with the untransformed data. In contrast, the REP transformation removes data points, treating zeros as missing values, and converts values ≥1 to 0.999. However, using the SV transformation resulted in slightly more error (simulations) or bias (real data) than did using the REP or no transformation.

The potential negative impacts of rapid increases in atmospheric CO_2_ require a better understanding of the critical role of litter decomposition in the global carbon cycle. This, in turn, requires accurate estimates of litter decomposition rates. Our results show that nonlinear beta regression is a useful method for estimating these rates. However, with the data explored to date, it did not often produce dramatically different results from standard nonlinear regression. Yet, given the type of heteroscedasticity found in most decomposition data, we suggest that the two methods should be considered alongside one another. Furthermore, our results suggest that regression method choice will have the smallest impacts during mid and late stage decomposition.

## Supporting Information

Figure S1 Minimum and maximum decomposition rates (*k*) versus total experiment time from the Adair et al. [3] single pool decomposition review.
(DOCX)

Figure S2 Histograms of sampling times as a proportion of total time from the Adair et al. [3] single pool decomposition review.
(DOCX)

Figure S3 Percent bias for simulations using beta error only with *k* estimated by each regression technique.
(DOCX)

Figure S4 Percent bias for simulations using beta error only with *k* estimated by each regression technique.
(DOCX)

Figure S5 Average *k* value for simulations using beta error only with *k* estimated by each regression technique.
(DOCX)

Figure S6 Percent bias for simulations using beta error with normal error (σ = 0.05) added with *k* estimated by each regression technique (SV transformation).
(DOCX)

Figure S7 Percent relative error for simulations using beta error with normal error (σ = 0.05) added with *k* estimated by each regression technique (SV transformation).
(DOCX)

Figure S8 Average *k* value for simulations using beta error with normal error (σ = 0.05) added with *k* estimated by each regression technique (SV transformation).
(DOCX)

Table S1 Bias and ΔAIC*_c_* values for Hobbie [14] and Laliberté and Tylianakis [12] data where normal *k* estimates were greater than beta *k* estimates.
(DOCX)

Appendix S1 R code to perform nonlinear beta regression.
(R) Click here for additional data file.
