# Supplementary material for: Estimating Litter Decomposition Rate in Single-Pool Models Using Nonlinear Beta Regression
Source: PLoS One. 2012 Sep 25;7(9):e45140. doi: 10.1371/journal.pone.0045140 (PMC3458010; doi:10.1371/journal.pone.0045140)

Figure S1. Minimum and maximum decomposition rates (*k*) versus total experiment time from the Adair et al. (2010) single pool decomposition review. These values are compared to the values chosen for the data simulation (gray lines).


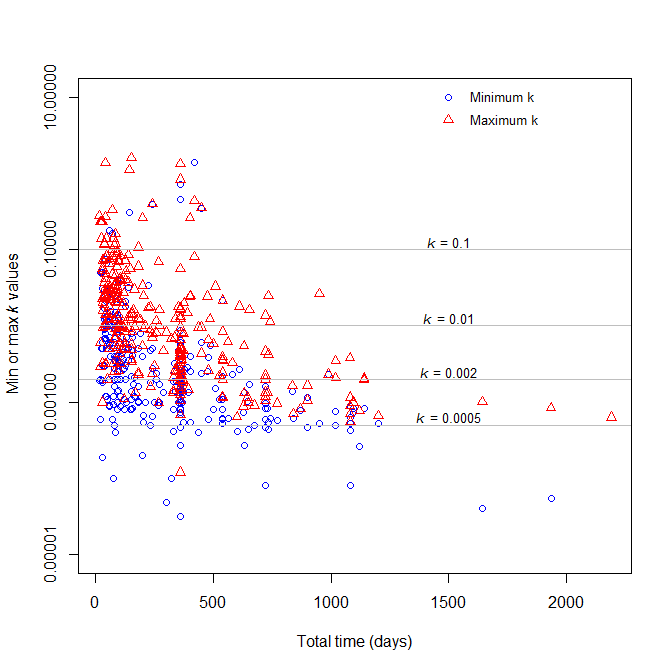

Supplement: Figure S1 — Minimum and maximum decomposition rates (k) versus total experiment time from the Adair et al. [3] single pool decomposition review. (DOCX) [file pone.0045140.s001.docx]
