# Supplementary material for: Estimating Litter Decomposition Rate in Single-Pool Models Using Nonlinear Beta Regression
Source: PLoS One. 2012 Sep 25;7(9):e45140. doi: 10.1371/journal.pone.0045140 (PMC3458010; doi:10.1371/journal.pone.0045140)

Figure S2. Histograms of sampling times as a proportion of total time from the Adair et al. (2010) single pool decomposition review. Panels show histograms from studies that used (a) two measurements, (b) five measurements, (c) seven measurements and (d) ten measurements. Asterisks indicate the measurement times chosen for the simulations with two, five, seven and ten measurements (the most common bins). For the simulations, the average of the selected bin measurements was used.


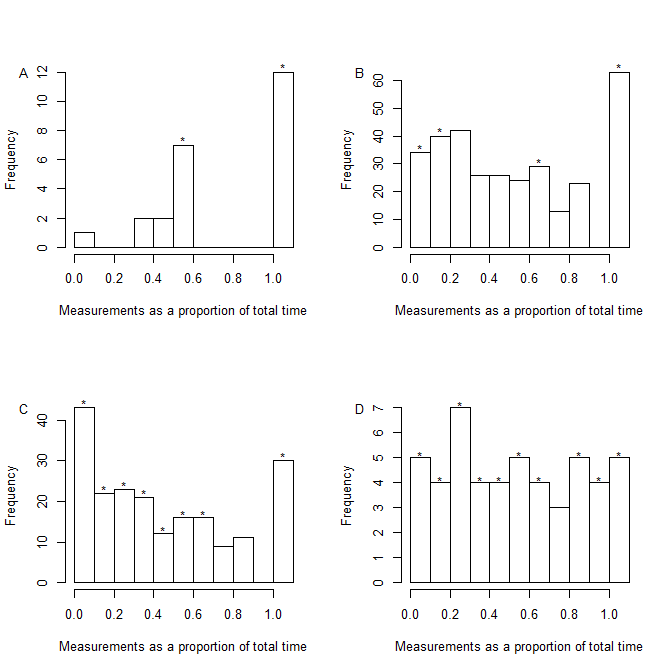

Supplement: Figure S2 — Histograms of sampling times as a proportion of total time from the Adair et al. [3] single pool decomposition review. (DOCX) [file pone.0045140.s002.docx]
