# Supplementary material for: Estimating Litter Decomposition Rate in Single-Pool Models Using Nonlinear Beta Regression
Source: PLoS One. 2012 Sep 25;7(9):e45140. doi: 10.1371/journal.pone.0045140 (PMC3458010; doi:10.1371/journal.pone.0045140)

Figure S5. Average *k* value for simulations using beta error only with *k* estimated by each regression technique: (a) *k* = 0.1, (b) *k* = 0.01, (c) *k* = 0.002 and (d) *k* = 0.0005. Blue dots = NLS, Red dots = Normal ML, gray/black dots = Beta ML.


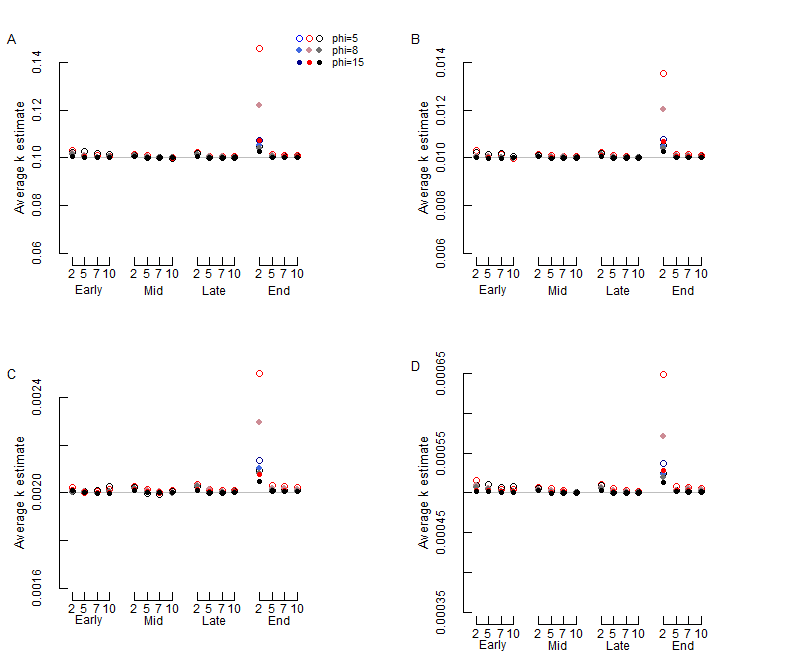

Supplement: Figure S5 — Average k value for simulations using beta error only with k estimated by each regression technique. (DOCX) [file pone.0045140.s005.docx]
