# Supplementary material for: Estimating Litter Decomposition Rate in Single-Pool Models Using Nonlinear Beta Regression
Source: PLoS One. 2012 Sep 25;7(9):e45140. doi: 10.1371/journal.pone.0045140 (PMC3458010; doi:10.1371/journal.pone.0045140)

Figure S6. Percent bias for simulations using beta error with normal error (σ = 0.05) added with *k* estimated by each regression technique (SV transformation): (a) *k* = 0.1, (b) *k* = 0.01, (c) *k* = 0.002 and (d) *k* = 0.0005. Blue dots = NLS, Red dots = Normal ML, gray/black dots = Beta ML. Gray lines show zero bias.


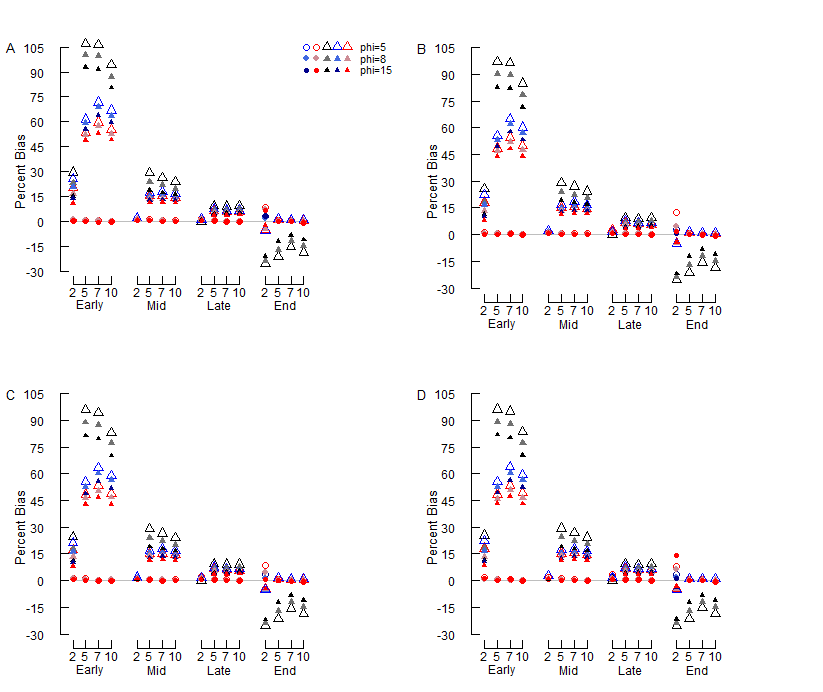

Supplement: Figure S6 — Percent bias for simulations using beta error with normal error (σ = 0.05) added with k estimated by each regression technique (SV transformation). (DOCX) [file pone.0045140.s006.docx]
