# Supplementary material for: Estimating Litter Decomposition Rate in Single-Pool Models Using Nonlinear Beta Regression
Source: PLoS One. 2012 Sep 25;7(9):e45140. doi: 10.1371/journal.pone.0045140 (PMC3458010; doi:10.1371/journal.pone.0045140)
Supplement: Table S1 — Bias and ΔAICc values for Hobbie [14] and Laliberté and Tylianakis [12] data where normal k estimates were greater than beta k estimates. (DOCX) [file pone.0045140.s009.docx]

**Table S1. Bias and ΔAIC*_c_* values for Hobbie (2008) and Laliberté and Tylianakis (2011) data where normal *k* estimates were greater than beta *k* estimates.**

|  | SV transformation | | | | | | Replacement transformation | | | | | |
| --- | --- | --- | --- | --- | --- | --- | --- | --- | --- | --- | --- | --- |
|  | Beta errors | | Normal errors | | ΔAIC*_c_* | | Beta errors | | Normal errors | | ΔAIC*_c_* | |
| Data | *k* | FB^1^/RB^2^ | *k* | FB^1^/RB^2^ | Beta | Normal | *k* | FB^1^/RB^2^ | *k* | FB^1^/RB^2^ | Beta | Normal |
| Laliberté & Tylianakis (2011) | | | | | | | | | | | | |
|  | 0.0084 | 0.6882 / 1.1236 | 0.0139 | 0.4862 / 0.6879 | 1.1 | 0 | 0.0112 | 0.1574 / 0.183 | 0.0139 | -0.0256 / -0.027 | 3.6 | 0 |
|  | 0.0086 | 0.5862 / 0.868 | 0.0120 | 0.4478 / 0.604 | 0 | 0.6 | 0.0105 | 0.1079 / 0.1194 | 0.0121 | -0.0061 / -0.0063 | 3.8 | 0 |
|  | 0.0078 | 0.5487 / 0.8632 | 0.0104 | 0.4281 / 0.6217 | 0 | 5.0 | 0.0089 | 0.1485 / 0.1831 | 0.0104 | 0.0299 / 0.0347 | 3.2 | 0 |
|  | 0.0085 | 0.4992 / 0.6934 | 0.0101 | 0.4298 / 0.5705 | 0 | 5.7 | 0.0095 | 0.0917 / 0.1002 | 0.0101 | 0.0435 / 0.0464 | 2.2 | 0 |
|  | 0.0081 | 0.4938 / 0.7372 | 0.0098 | 0.4122 / 0.5839 | 0 | 3.6 | 0.0089 | 0.1139 / 0.1358 | 0.0099 | 0.0329 / 0.0377 | 3.8 | 0 |
|  | 0.0078 | 0.4744 / 0.6863 | 0.0089 | 0.4162 / 0.58 | 0 | 9.6 | 0.0086 | 0.1018 / 0.1183 | 0.0089 | 0.0718 / 0.0822 | 0 | 0.7 |
| Hobbie (2008) | | | | | | | | | | | | |
|  | 0.0016 | 0.0855 / 0.0691 | 0.0020 | -0.0529 / -0.0399 | 0 | 69.6 | 0.0014 | 0.1959 / 0.168 | 0.0019 | -0.0329 / -0.025 | 0 | 92.0 |
|  | 0.0016 | 0.1073 / 0.0865 | 0.0020 | -0.0299 / -0.0225 | 0 | 82.3 | 0.0015 | 0.1792 / 0.1501 | 0.0019 | -0.0222 / -0.0168 | 0 | 98.0 |
|  | 0.0016 | 0.0757 / 0.0606 | 0.0019 | -0.0385 / -0.0291 | 0 | 75.4 | 0.0014 | 0.1948 / 0.1663 | 0.0019 | -0.0261 / -0.0198 | 0 | 100.9 |
|  | 0.0016 | 0.0756 / 0.065 | 0.0019 | -0.0451 / -0.0365 | 0 | 55.5 | 0.0014 | 0.2059 / 0.1899 | 0.0019 | -0.0359 / -0.0292 | 0 | 79.0 |
|  | 0.0015 | 0.1282 / 0.2509 | 0.0019 | -0.0412 / -0.074 | 13.9 | 0 | 0.0015 | 0.1282 / 0.2509 | 0.0019 | -0.0412 / -0.074 | 13.9 | 0 |
|  | 0.0016 | 0.0705 / 0.0606 | 0.0018 | -0.037 / -0.0302 | 0 | 68.7 | 0.0015 | 0.1153 / 0.1015 | 0.0018 | -0.0327 / -0.0267 | 0 | 75.2 |
|  | 0.0014 | -0.0672 / -0.0986 | 0.0018 | -0.2265 / -0.3086 | 10.3 | 0 | 0.0012 | 0.0762 / 0.1202 | 0.0014 | -0.0362 / -0.0539 | 10.3 | 0 |
|  | 0.0016 | 0.0725 / 0.062 | 0.0018 | -0.0261 / -0.0212 | 0 | 70.5 | 0.0016 | 0.0602 / 0.0512 | 0.0018 | -0.0231 / -0.0189 | 0 | 63.9 |
|  | 0.0015 | 0.0249 / 0.022 | 0.0017 | -0.0417 / -0.0356 | 0 | 63.5 | 0.0015 | 0.0228 / 0.0202 | 0.0017 | -0.0402 / -0.0344 | 0 | 61.0 |
|  | 0.0015 | 0.031 / 0.0271 | 0.0017 | -0.034 / -0.0288 | 0 | 77.5 | 0.0015 | 0.0239 / 0.0208 | 0.0017 | -0.0313 / -0.0265 | 0 | 73.0 |
|  | 0.0015 | 0.0155 / 0.0144 | 0.0016 | -0.0312 / -0.0283 | 0 | 59.1 | 0.0015 | 0.0138 / 0.0127 | 0.0016 | -0.0296 / -0.0268 | 0 | 56.7 |
|  | 0.0014 | 0.1006 / 0.1948 | 0.0016 | -0.0014 / -0.0025 | 10.9 | 0 | 0.0014 | 0.1006 / 0.1948 | 0.0016 | -0.0014 / -0.0025 | 10.9 | 0 |
|  | 0.0015 | 0.0578 / 0.0983 | 0.0016 | -0.0053 / -0.0086 | 8.3 | 0 | 0.0015 | 0.0578 / 0.0983 | 0.0016 | -0.0053 / -0.0086 | 8.3 | 0 |
|  | 0.0015 | 0.0028 / 0.0025 | 0.0016 | -0.0391 / -0.035 | 0 | 57.8 | 0.0014 | 0.0413 / 0.0385 | 0.0016 | -0.0341 / -0.0306 | 0 | 61.3 |
|  | 0.0013 | 0.0193 / 0.0317 | 0.0016 | -0.1078 / -0.1664 | 7.6 | 0 | 0.0011 | 0.1327 / 0.2312 | 0.0014 | -0.0169 / -0.0273 | 13.2 | 0 |
|  | 0.0015 | 0.0026 / 0.004 | 0.0015 | 0.004 / 0.0062 | 24.2 | 0 | 0.0015 | 0.0026 / 0.004 | 0.0015 | 0.004 / 0.0062 | 24.2 | 0 |
|  | 0.0014 | 0.0099 / 0.0136 | 0.0015 | -0.0224 / -0.0302 | 3.0 | 0 | 0.0014 | 0.0099 / 0.0136 | 0.0015 | -0.0224 / -0.0302 | 3.0 | 0 |
|  | 0.0014 | 0.0515 / 0.0884 | 0.0015 | -0.007 / -0.0117 | 6.3 | 0 | 0.0014 | 0.0515 / 0.0884 | 0.0015 | -0.007 / -0.0117 | 6.3 | 0 |
|  | 0.0013 | 0.0446 / 0.0416 | 0.0015 | -0.0508 / -0.0452 | 0 | 72.0 | 0.0012 | 0.1271 / 0.1239 | 0.0015 | -0.0431 / -0.0385 | 0 | 94.3 |
|  | 0.0014 | 0.0463 / 0.0813 | 0.0015 | -0.0051 / -0.0088 | 7.4 | 0 | 0.0014 | 0.0463 / 0.0813 | 0.0015 | -0.0051 / -0.0088 | 7.4 | 0 |
|  | 0.0013 | 0.0915 / 0.2159 | 0.0015 | 0.0174 / 0.0395 | 14.9 | 0 | 0.0013 | 0.0915 / 0.2159 | 0.0015 | 0.0174 / 0.0395 | 14.9 | 0 |

^1^FB = fractional bias.

^2^RB = relative bias.
